# Population structure and genetic diversity of Mi pigs based on SINE-RIPs

**DOI:** 10.3389/fvets.2025.1500115

**Published:** 2025-04-24

**Authors:** Xiaoyan Wang, Chenyu Zhou, Yao Zheng, Miao Yu, Jia He, Cai Chen, Suwei Qiao, Ali Shoaib Moawad, Guoxing Tian, Bixia Li, Chengyi Song

**Affiliations:** ^1^College of Animal Science and Technology, Yangzhou University, Yangzhou, China; ^2^International Joint Research Laboratory in Universities of Jiangsu Province of China for Domestic Animal Germplasm Resources and Genetic Improvement, Yangzhou, China; ^3^Department of Animal Production, Faculty of Agriculture, Kafrelsheikh University, Kafrelsheikh, Egypt; ^4^Jintan Mi Pig Breeding Farm, Changzhou City, Jiangsu, China; ^5^Institute of Animal Science, Jiangsu Academy of Agricultural Sciences, Nanjing, China

**Keywords:** Mi pig, retrotransposon insertion polymorphisms, SINE, genetic diversity, population structure

## Abstract

Mi pigs, a Chinese native breed found in Jintan and Yangzhong in Jiangsu Province, were recorded as having only a few hundred members in the latest national livestock and poultry genetic resources survey. To explore their conservation and breeding prospects, 18 SINE Retrotransposon Insertion Polymorphisms (sine-rips) were analyzed using PCR to assess the population structure and genetic diversity of Mi pigs. These pigs were grouped into eight families based on a UPGMA phylogenetic tree. The genetic distances between the Mi pig populations and commercial breeds ranged from 0.3712 to 0.7609, indicating significant divergence. Conversely, they showed a closer genetic relationship with other local Jiangsu breeds, with distances varying from 0.0943 to 0.6122, a finding supported by the UPGMA tree. The populations displayed a substantial degree of outbreeding, with Fis values from −0.4744 (M5) to −0.0847 (M8) and Fst values from 0.0534 (M3, M8) to 0.2265 (M2, M7), highlighting their genetic diversity which is crucial for the conservation of Mi pigs. Despite this diversity, the population sizes were uneven, with M5, M7, and M8 having 6, 5, and 7 individuals, respectively. These findings lay a theoretical foundation for the ongoing conservation and breeding efforts for Mi pigs.

## 1 Introduction

**Figure 1:**
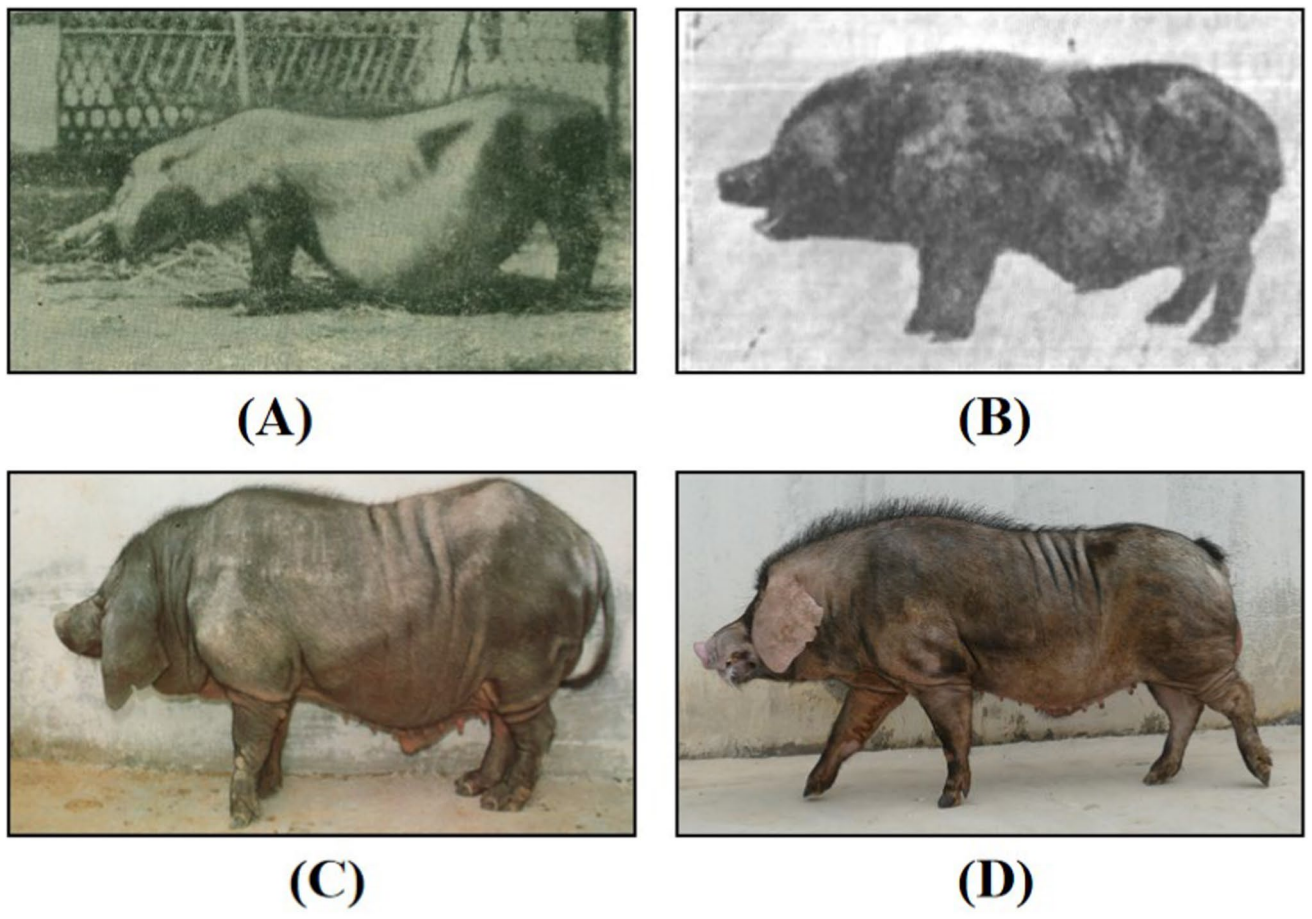
Photos of Mi pigs. **(A)** Photo of Mi pig taken by Zhang (48) in 1956; **(B)** photo of Mi pig taken by Cao (2) in 1982; **(C)** photo of Mi pig in 1991 (48); **(D)** photo of Mi pig in 2023. Reproduced with permission.

Mi pigs, a native breed from Jiangsu Province, specifically found in Jintan and Yangzhong, are characterized by their distinct sharp snouts and hindquarters, and round bellies, which resemble grains (Figure 1). They are renowned for their high fertility, strong maternal instincts, and robust adaptability. Mi pigs are believed to have originated from crossbreeding between local pigs from Jintan and Yangzhong and Huai pigs several centuries ago. The Huai pig, an ancient breed from the Huaibei plain, is even highlighted in the historical Compendium of Materia Medica (1). Due to wars and natural disasters, residents of the Huaibei plain migrated to central Jiangsu Province, bringing Huai pigs with them. During this period, Mi pigs thrived in the region, with the population peaking at 45,000 in Jintan and Yangzhong counties by the late 1980s (2). However, recent data from the third national survey on livestock and poultry genetic resources (2021–2023) show that only a few hundred Mi pigs remain. Crossbreeding between Mi pigs and local varieties has led to the development of Erhualian and Hongdenglong pigs, which are now collectively recognized as part of the Taihu pig population (3–5).

Since the 1980s, the introduction of numerous Western commercial pig breeds to China has led to a significant decline in the populations of most Chinese native pig breeds, due to their slower growth rates and lower lean meat percentages. The Mi pig population has been similarly affected, with only a few hundred individuals currently maintained at the Jintan Mi Pig Breeding Farm. The spread of African swine fever in China has introduced a severe health risk to pigs and heightened the risk of extinction for this population. Despite these challenges, native pigs offer a safeguard against potential future issues such as long-term climate change effects or the emergence of new diseases (6). As foundational elements for developing new breeds and preserving biodiversity, the conservation of these native pigs is gaining increasing focus. Consequently, research into the genetic diversity of Mi pigs is crucial as it provides essential insights for their ongoing conservation and breeding efforts.

Numerous molecular markers based on sequence variations in the pig genome are used to assess genetic diversity, including SNPs, SVs, and Microsatellites. In a previous study, we developed a marker system that utilizes retrotransposon insertion polymorphisms (RIPs) to analyze population structure and genetic diversity in pigs. Retrotransposons, a predominant type of transposon, comprise about 40% of the pig genome (7–9). These elements are capable of moving within the genome through a “copy-and-paste” mechanism, playing a significant role in the evolution of mammalian genomes (10). Because of their copy-paste mechanism during mobilization, retrotransposons generate plentiful polymorphism throughout the genomes. Retrotransposons cannot excise themselves from their insertion locations and this unidirectionality of integration confers great advantages in reconstructing pedigrees and phylogenies because the ancestral state is obvious compared with almost all other genetic polymorphisms (11).

Retrotransposons can be divided into two major categories: Long terminal repeats (LTR) and Non-long terminal repeats (non-LTR). LTR primarily includes endogenous retroviruses (ERVs), while the non-LTR category predominantly comprises Short Interspersed Nuclear Elements (SINEs) and Long Interspersed Nuclear Elements (LINEs). SINEs, derived from tRNA, are widely distributed in eukaryotic genomes and accounted for 11.05% of the sequenced pig genome (8). They typically range from 150 to 300 bp in length and consist of a 5′ head, a main body, and a 3′ tail. SINEs are evolutionarily removed from genomes at a slower rate compared to LINEs and LTRs, which are present as larger fragments. SINE insertions can positively regulate gene expression, enhance the diversity of gene isoforms, and contribute to the creation of long noncoding RNAs (12). Therefore, SINEs, which believed to be more tolerable for hosts compared with LTRs and LINEs, can co-evolve with host genomes, and can exert a wider impact on the shaping of genes and on genome evolution (10). We have identified several SINEs that act as enhancers or repressors, influencing gene expression and even the phenotypes of pigs (13–16).

Reversed Transposon Insertion Polymorphism (RIP) denotes the occurrence or absence of reversed transposon insertions at particular genomic sites within a species population. This significance stems not only from the increasing number of involved loci and sequences but also from their complex and profound effects on genome structure and gene functionality. Transposon-based labeling systems, which are readily identifiable via PCR, are widely used in phylogenetic analysis, genetic diversity evaluation, breeding programs, and mapping studies across various crops and trees.

Most SINE RIPs are regarded as neutral markers of identical descent and are beneficial for aligning gene trees with species trees, minimizing phylogenetic errors (17). These markers, based on SINE insertions, have been extensively utilized in plant genotyping for variety identification and molecular breeding (18–21). Similarly, in animal genetics, RIPs are increasingly being explored as species- or breed-specific markers. Four SINE RIPs were identified as human-specific insertions and trace human roots to Africa (22). SINE insertions gave important information for distinguishing a clear European origin in Eldorado A (23). Using SINE RIPs, whales, ruminants and hippopotamuses form a monophyletic group (24). They also can effectively distinguish genetic differences among six populations of *Coilia nasus* from the Yangtze River Basin (25–27). These studies highlight RIPs’ potential in verifying breed purity and assisting marker-assisted selection for desirable traits.

Additionally, this molecular system is rapid, robust, and cost-effective in practice. In previous research, we identified over 35,000 SINE RIP markers in pig genomes. Young SINE elements are instrumental in introducing new genetic variations and influencing the evolution of the pig genome (28). We have developed an effective marker system using young SINE RIPs, which has been applied to analyze genetic diversity and population structure in various pig populations including miniature pigs (29), native pigs in Jiangsu province (30), even crossing breeding Sujiang pigs (31). Considering the urgency and importance of conservation of Mi pigs, we have employed this molecular marker to evaluate the genetic diversity for better conservation and continuous breeding of Mi pigs.

## 2 Materials and methods

### 2.1 Samples

Ear samples were collected from 134 individuals at the Jintan Mi Pig Breeding Farm in Jiangsu Province. Additionally, to analyze genetic diversity and population structure, 183 samples were gathered from six different breeds. This included 32 samples each from Largewhite (LW), Landrace (LD), and Duroc (DRC) breeds (all from Anqing, Anhui), 32 from Erhualian pigs (EHL, Changzhou, Jiangsu), 23 from Huai pigs (H, Donghai, Jiangsu), and 32 from Hongdenglong pigs (HDL, Liyang, Jiangsu).

### 2.2 DNA extraction

Genomic DNA was extracted from the ear tissue samples following the instruction provided with the DNA extraction kit (Tiangen, Beijing). The concentration and quality of the DNA were assessed using a NanoPhotometer (Implen, Munich, Germany). Subsequently, the DNA samples were stored at −20°C.

### 2.3 Primers, PCR and gel electrophoresis

The 18 SINE-RIPs previously identified in Chinese native pigs were selected for genotyping analysis (29–31). The details of specific primers are presented in Supplementary Table S1. The primers were synthesized by Nanjing TSINGKE Biotechnology Co., Ltd. PCR reaction system contained 10 μL 2 × Taq Master Mix, 1 μL DNA, 1 μL Primer F, 1 μL Primer R and 7 μL ddH2O. The PCR products were detected by electrophoresis using 1.5% agarose gel (TSINGKE, Nanjing, China), followed by 10 min of ethidium bromide (EB) staining and genotyping using a UV fluorescence system (Tanon, Shanghai, China). Three genotypes were identified for the SINE RIPs: a single larger band of PCR fragments indicated a homozygote for SINE insertion, a single smaller band indicated a homozygote without SINE insertion, and two bands including one larger and one smaller band indicated a heterozygote.

### 2.4 Data analysis

The effective number of alleles (Ne), observed heterozygosity (Ho), expected heterozygosity (He), *F*-value, and Hardy–Weinberg equilibrium were assessed using Popgene software (Version 1.32) (32). The polymorphic information content (PIC) was determined using the following formula, where Pi and Pj are the allele frequencies at alleles i and j respectively, and m is the number of alleles (33).


$$\mathrm{PIC}=1-\overset{m}{\underset{i=1}{\sum }}Pi^{2}-\overset{m-1}{\underset{i=1}{\sum }}\overset{m}{\underset{j=i+1}{\sum }}2Pi^{2}Pj^{2}$$

Cluster analysis was conducted using Nei’s genetic distance, and an UPGMA (Unweighted Pair Group Method with Arithmetic Mean) phylogenetic tree was generated using Mega7 software (34). Principal Component Analysis (PCA) analysis was performed using OriginPro software (35). The population structure of the seven pig breeds was constructed using the Bayesian method implemented in Structure software.

## 3 Results

### 3.1 Identification of SINE-RIPs and population structure in Mi pigs

**Figure 2:**
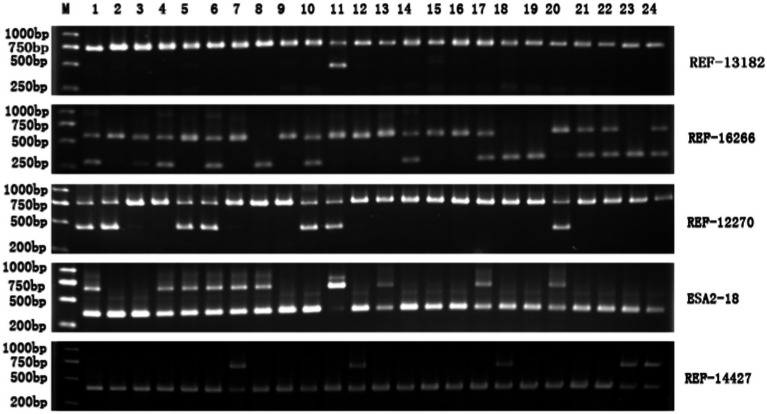
Representative electropherogram of SINE-RIPs in the Mi pig population. M: DL2000 DNA marker; 1–24: 24 individuals; the right side shows the names of the SINE-RIPs loci.

**Figure 3:**
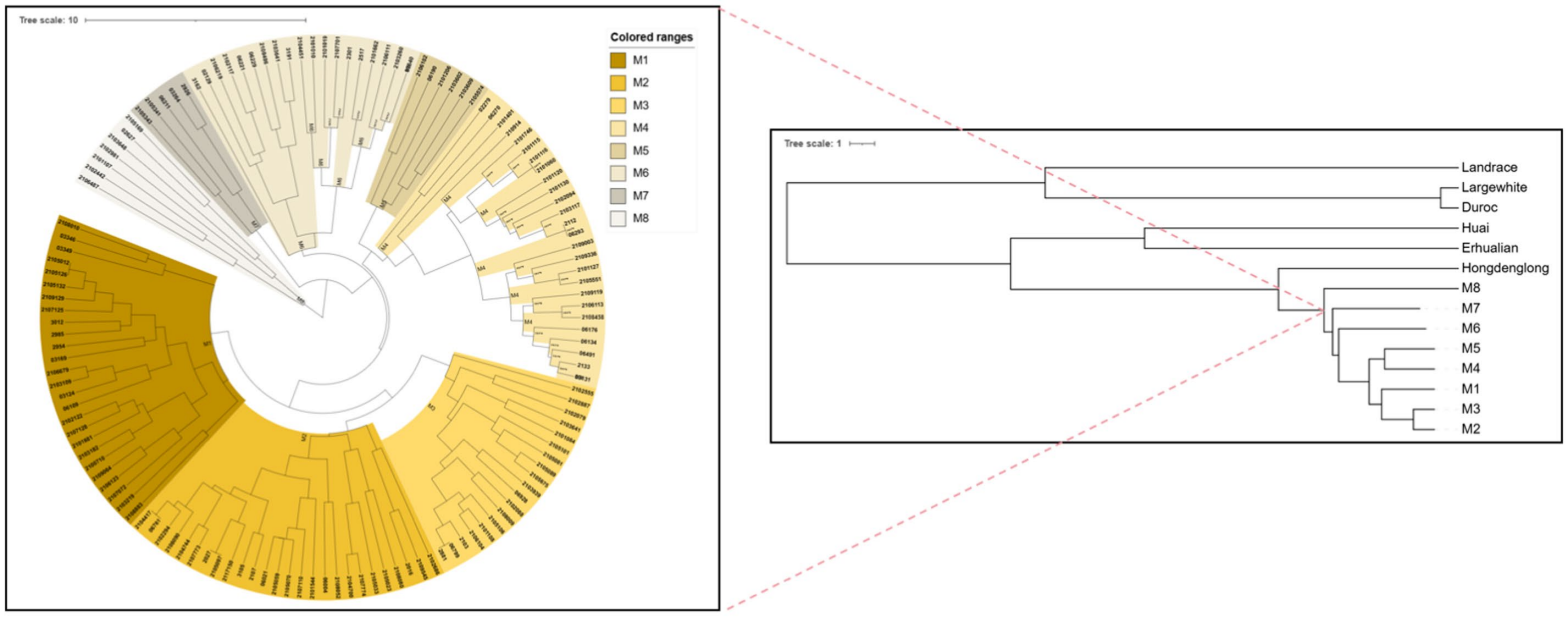
UPGMA tree for cluster analysis with Mi pigs and the other six germplasm individuals based on Nei’s genetic distance and analysis of population structure in Mi pigs based on UPGMA tree. The number of individuals included in each family, as well as the ID of male breed indicated by the black signs.

The 18 SINE-RIPs in the Mi pigs were detected allele by allele through PCR and electrophoresis. The representative electropherogram displaying the SINE-RIPs is shown in Figure 2. Three distinct genotypes—SINE+/+, SINE−/−, and SINE+/− could be easily distinguished by the number of bands and the length of the fragments. Based on the UPGMA tree (Figure 3), the 134 Mi pigs were categorized into 8 clusters, with each cluster containing one to three boars, reflecting their geographical origins. These clusters will be referred to as M1 through M8 in subsequent chapters.

### 3.2 Genetic diversity among Mi populations and other pig breeds

Among all RIPs, as shown in Table 1, REF-13182 and REF-9435 did not meet Hardy– Weinberg equilibrium across nine populations, while ESA1-98, REF-17668, and ESA1-33 were out of equilibrium in seven populations. REF-14427, REF-3992, and REF-5597 failed to meet Hardy–Weinberg equilibrium in six populations, with fewer deviations observed at other loci. Moreover, out of the 18 polymorphic loci analyzed, most populations exhibited polymorphism for SINE-RIPs. Notably, REF-13182 and REF-9435 showed the least polymorphism, evident in only five populations. In contrast, REF-2929 and REF-10096 were polymorphic across 14 populations. The remaining loci varied, with 6–13 populations displaying polymorphism. Among the breeds, Duroc pigs exhibited the least polymorphism with 17 loci showing no variation, followed by Large White pigs at 11 loci, the M7 population at 9 loci, M2 pigs at 8 loci, Landrace pigs at 7 loci, and M4 pigs at 6 loci. Other local pig breeds and the Mi population generally exhibited polymorphism at 0–5 loci (Table 1).

**Table 1:** Analysis of SINE-RIPs polymorphism in Mi pig.

RIP	Insertion frequency															Number of populations without polymorphism	Number of populations with polymorphism/number of populations with Hardy–Weinberg imbalance violation
	LD	LW	DRC	EHL	H	HDL	M(average)	M1	M2	M3	M4	M5	M6	M7	M8		
ESA1-98	1.00	–	–	1.00	–	0.20	0.36	0.10	0.75	0.26	–	0.58	0.24	–	0.21	7	8/0
REF-12270	0.44	0.45	–	0.48	–	0.77	0.68	0.96	0.64	0.43	0.71	1.00	0.89	0.70	0.14	3	12/4
REF-13182	–	0.08	0.19	1.00	1.00	0.83	0.99	1.00	1.00	1.00	0.98	1.00	1.00	1.00	0.93	9	6/0
REF-14427	–	–	–	0.58	0.96	0.52	0.26	0.10	–	0.37	0.31	0.42	0.11	–	–	6	9/2
REF-16131	–	–	–	0.39	0.22	–	0.63	0.21	0.72	0.63	0.71	0.83	0.71	0.75	0.50	4	11/3
REF-16266	–	–	–	0.08	–	0.81	0.62	0.70	0.69	0.69	0.23	0.50	0.61	0.70	0.86	4	11/2
REF-17668	–	–	–	0.39	–	–	0.10	0.08	0.03	0.07	–	0.33	0.03	–	0.07	7	8/1
ESA2-58	0.67	0.03	–	0.92	0.43	0.61	0.40	0.19	0.19	0.13	0.56	0.33	–	1.00	–	3	12/2
REF-21609	0.13	-	–	0.05	0.13	0.55	0.54	0.62	0.53	0.67	0.24	0.33	0.29	0.90	0.71	2	13/3
REF-2929	0.44	0.03	–	0.84	0.67	0.59	0.53	0.65	0.67	0.56	0.54	0.67	0.39	0.30	0.43	1	14/6
ESA1-16	1.00	0.16	–	0.98	0.37	0.81	0.82	1.00	1.00	0.94	0.94	0.58	0.82	0.80	0.50	4	11/0
REF-3992	0.63	–	–	0.59	1.00	0.58	0.11	0.02	–	0.06	–	–	0.21	–	0.14	6	9/1
REF-5597	–	0.03	–	0.52	-	0.91	0.88	0.88	1.00	0.96	1.00	0.83	0.64	1.00	0.71	6	9/1
ESA2-18	0.64	–	–	0.66	0.43	0.64	0.15	0.12	0.03	0.06	0.38	0.17	0.21	0.10	0.14	2	13/1
ESA1-33	1.00	–	–	0.23	0.39	0.09	0.18	0.04	–	0.07	–	–	0.24	–	0.36	7	8/1
REF-9435	–	–	–	0.84	0.61	0.81	0.99	1.00	1.00	0.98	1.00	1.00	1.00	1.00	0.93	9	6/0
REF-10096	0.08	0.08	–	0.53	0.89	0.53	0.38	0.31	0.03	0.41	0.52	0.50	0.66	0.50	0.14	1	14/6
REF-11062	0.40	–	–	0.45	0.63	0.80	0.91	0.96	1.00	0.89	0.98	0.92	0.89	0.80	0.86	3	12/1
Number of Non-Polymorphic Sites in a Population	7	11	17	0	5	2	–	–	8	1	6	5	3	9	2	–	–

The genetic parameters of Mi population and six breeds generated by 18 SINE-RIPs have been presented in Table 2. The Ne values of Mi populations ranged from 1.2863 (M7) to 1.5207 (M5), while the Fis values range from −0.4744 (M5) to −0.0847 (M8). The He values were between 0.6835 (M5) to 0.8240 (M2), and the Ho values ranged from 0.5556 (M5) to 0.7778 (M7). For other pig breeds, the Ne values ranged from 1.0243 (DRC) to 1.5699 (HDL), and the Fis values varied from 0.2308 (DRC) to 0.0070 (EHL). The He values were between 0.6645 (HDL) to 0.9828 (DRC), and the Ho values were from 0.6337 (HDL) to 0.9792 (DRC). The average PIC among 14 populations from 7 breeds was 0.1833, ranging from 0.2690 (HDL) to 0.0331 (DRC). Based on the PIC values, the Huai pig (H), HDL, and M8 were moderately polymorphic, while the remaining populations were lowly polymorphic.

**Table 2:** Genetic parameters of Mi populations and six breeds by 18 SINE-RIPs.

Breeds or population	Number	Ho	He	Ne	Fis	PIC
LD	32	0.7760 ± 0.3120	0.8191 ± 0.2262	1.3249 ± 0.4257	−0.2192 ± 0.3907	0.1509 ± 0.1691
LW	32	0.9323 ± 0.1190	0.9306 ± 0.1312	1.1039 ± 0.2387	−0.0387 ± 0.1027	0.0745 ± 0.1191
DRC	32	0.9792 ± 0.0884	0.9828 ± 0.0730	1.0243 ± 0.1033	−0.2308 ± 0.0000	0.0331 ± 0.0982
EHL	32	0.6892 ± 0.2347	0.6805 ± 0.1999	1.5687 ± 0.4102	0.0070 ± 0.2802	0.2532 ± 0.1393
H	23	0.7536 ± 0.2400	0.7643 ± 0.2243	1.4123 ± 0.4214	−0.0708 ± 0.1428	0.1912 ± 0.1940
HDL	32	0.6337 ± 0.1993	0.6645 ± 0.1643	1.5699 ± 0.3446	−0.1056 ± 0.2261	0.2690 ± 0.1149
M1	26	0.7617 ± 0.2191	0.7975 ± 0.1690	1.3068 ± 0.3063	−0.1428 ± 0.1570	0.1767 ± 0.1293
M2	19	0.7716 ± 0.2889	0.8240 ± 0.2117	1.2936 ± 0.3742	−0.2421 ± 0.2240	0.1488 ± 0.1602
M3	27	0.6847 ± 0.2789	0.7514 ± 0.1861	1.4073 ± 0.3738	−0.1890 ± 0.2301	0.2075 ± 0.1323
M4	26	0.6674 ± 0.3397	0.7666 ± 0.2232	1.4069 ± 0.4144	−0.3504 ± 0.2472	0.1901 ± 0.1643
M5	6	0.5556 ± 0.3524	0.6835 ± 0.2274	1.5207 ± 0.4088	−0.4744 ± 0.2549	0.2351 ± 0.1520
M6	19	0.6363 ± 0.2604	0.7150 ± 0.1773	1.4562 ± 0.3250	−0.2617 ± 0.2324	0.2326 ± 0.1297
M7	5	0.7778 ± 0.3059	0.8058 ± 0.2169	1.2863 ± 0.3428	−0.2231 ± 0.5003	0.1532 ± 01545
M8	7	0.6905 ± 0.2558	0.7027 ± 0.1729	1.4500 ± 0.3349	−0.0847 ± 0.4641	0.2501 ± 0.1064
Average	-	0.7364 ± 0.1095	0.7777 ± 0.0895	1.3666 ± 0.1534	−0.1876 ± 0.1237	0.1833 ± 0.0655

**Figure 4:**
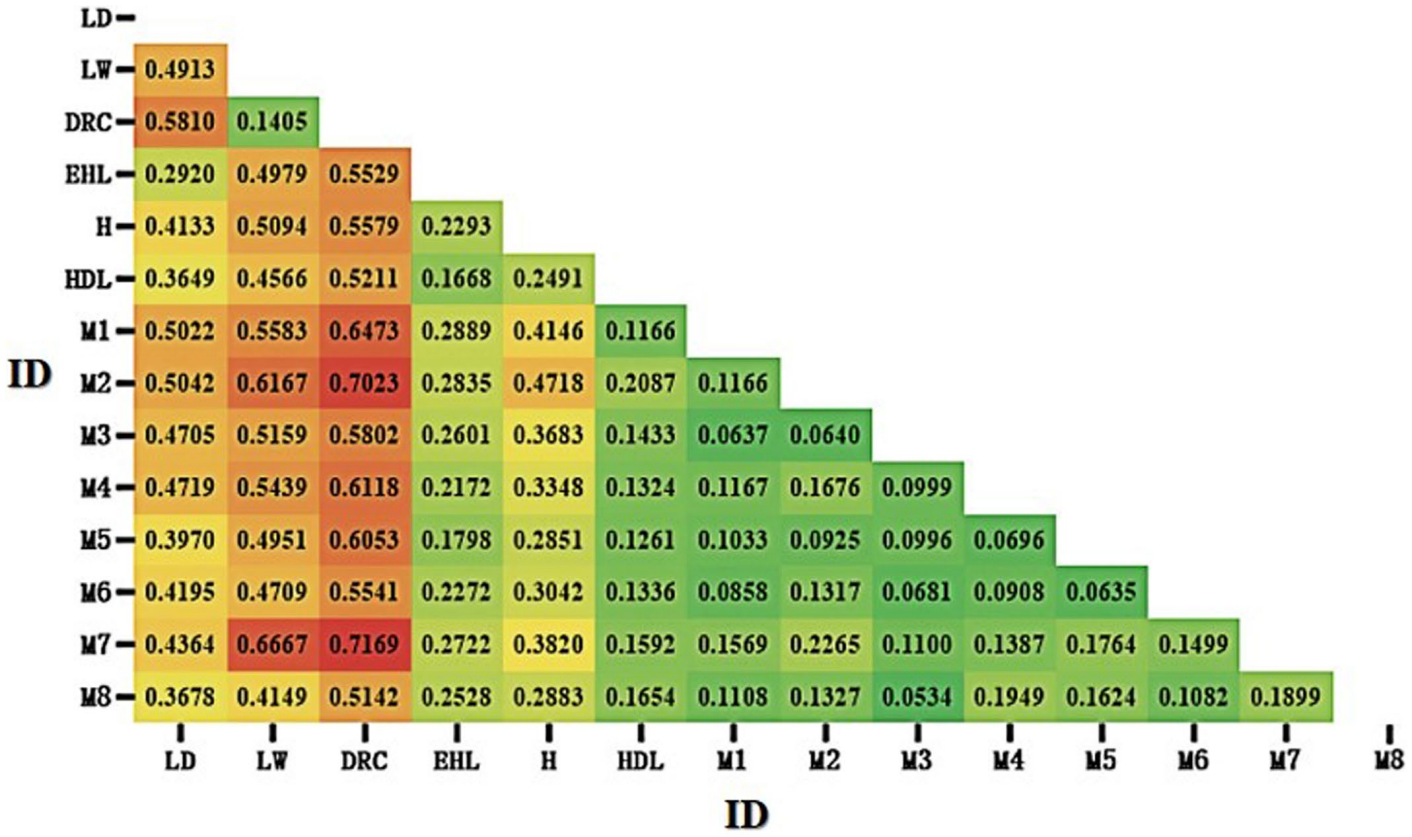
Heatmap of fixation index (Fst) among Mi pig families and six breeds of pigs. LD, Landrace; LW, Large White; DRC, Duroc; EHL, Erhualian; HDL, Hongdenglong; M, Mi; H, Huai (Red indicated higher Fst values, while green indicate lower Fst values).

The Fst heatmap generated by Popgene32 (version 1.32) among Mi population and six breeds has been displayed in Figure 4. The three commercial pig breeds (Landrace, Large White, and Duroc) were highly differentiated from other local pig populations. In contrast, the Fst values among Jiangsu local pigs (Erhualian, Huai, Hongdenglong, and Mi pigs), including Mi pig families, were relatively low, indicating a smaller degree of differentiation and similar genetic backgrounds. Among the 14 populations of the seven pig breeds, the Fst value between M7 and Duroc pigs was the highest (0.7169), indicating the greatest degree of population differentiation. The Fst values between Mi families ranged from 0.0534 to 0.2265, indicating the smallest degree of population differentiation compared with other Jiangsu local breeds and commercial pigs.

### 3.3 Genetic distances

Detailed information on Nei’s genetic identity and genetic distances between Mi pig families and other pig breeds were presented in Table 3. Among the seven breeds, the genetic distances between Jiangsu local populations and commercial breeds were greater than those between the local populations themselves. The range of genetic distances varied from 0.0143 to 0.7609, with the smallest genetic distance (0.0143) observed between Duroc and Large White, and the largest genetic distance (0.7609) noted between Landrace and M8.

**Table 3:** Nei’s genetic identity and genetic distances between Mi populations and six breeds.

ID	LD	LW	DRC	EHL	H	HDL	M1	M2	M3	M4	M5	M6	M7	M8
LD	–	0.7306	0.7039	0.7336	0.6303	0.6116	0.5299	0.5509	0.5232	0.5320	0.5043	0.5614	0.4673	0.5718
LW	0.3139	–	0.9858	0.5368	0.6484	0.5898	0.6240	0.5709	0.6163	0.6123	0.5860	0.6501	0.5607	0.6899
DRC	0.3511	0.0143	–	0.5171	0.6674	0.5530	0.5794	0.5483	0.6028	8.5792	0.5445	0.6104	0.5326	0.6842
EHL	0.3098	0.6221	0.6596	–	0.7769	0.8095	0.7170	0.7504	0.7261	0.7964	0.8049	0.7460	0.6935	0.7195
H	0.4616	0.4332	0.4043	0.2525	–	0.7474	0.6156	0.5422	0.6386	0.6996	0.6417	0.6975	0.5814	0.6981
HDL	0.4917	0.5280	0.5925	0.2113	0.2911	–	0.9100	0.8255	0.8670	0.8821	0.8437	0.8638	0.8591	0.8735
M1	0.6351	0.4716	0.5458	0.3326	0.4851	0.0943	–	0.9378	0.9619	0.9283	0.9169	0.9428	0.9073	0.9552
M2	0.5961	0.5605	0.6008	0.2872	0.6122	0.1918	0.0642	–	0.9656	0.8976	0.9318	0.9146	0.8793	0.9437
M3	0.6478	0.4840	0.5062	0.3201	0.4484	0.1427	0.0388	0.0350	–	0.9312	0.9199	0.9489	0.9243	0.9833
M4	0.6311	0.4905	0.5460	0.2276	0.3573	0.1255	0.0744	0.1080	0.0713	–	0.9320	0.9329	0.9213	0.9216
M5	0.6846	0.5345	0.6079	0.2170	0.4436	0.1700	0.0868	0.0706	0.0835	0.0704	–	0.9415	0.8755	0.9101
M6	0.5773	0.4306	0.4937	0.2931	0.3602	0.1464	0.0589	0.0893	0.0524	0.0695	0.0603	–	0.8746	0.9503
M7	0.7609	0.5786	0.6300	0.3660	0.5424	0.1519	0.0973	0.1287	0.0787	0.0819	0.1329	0.1340	–	0.9030
M8	0.5589	0.3712	0.3795	0.3292	0.3593	0.1353	0.0458	0.0579	0.0168	0.0817	0.0942	0.0510	0.1021	–

“–” indicated diagonal. Nei’s genetic identity is presented above the diagonal, and genetic distance is presented below the diagonal. LD, Landrace; LW, Large White; DRC, Duroc; EHL, Erhualian; HDL, Hongdenglong; M1-8, Mi population; H, Huai pig.

### 3.4 UPGMA tree and PCA plots

Based on the genetic distance of Nei’s among 134 Mi pigs, 32 Landrace, Large White, Duroc, Erhualian, Hongdenglong, and 23 Huai pigs, the pigs were divided into 14 branches. Additionally, a UPGMA tree generated based on the genetic distance of Nei’s among the seven breeds has been shown in Figure 3. The seven pig breeds were divided into two main branches, with commercial pig breeds forming one branch and Jiangsu local breeds forming the other. Among the commercial pig breeds, Large White and Duroc pigs formed a sub-branch, while Landrace pigs constituted a separate sub-branch. Within the Jiangsu local pig breeds, Huai and Erhualian pigs grouped into one sub-branch, and Hongdenglong and Mi populations formed another sub-branch. Within the Mi pig families, M7 formed a separate sub-branch, while M1, M2, and M3 along with M8 grouped into a smaller sub-branch, and M4, M5 along with M6 formed another small sub-branch.

**Figure 5:**
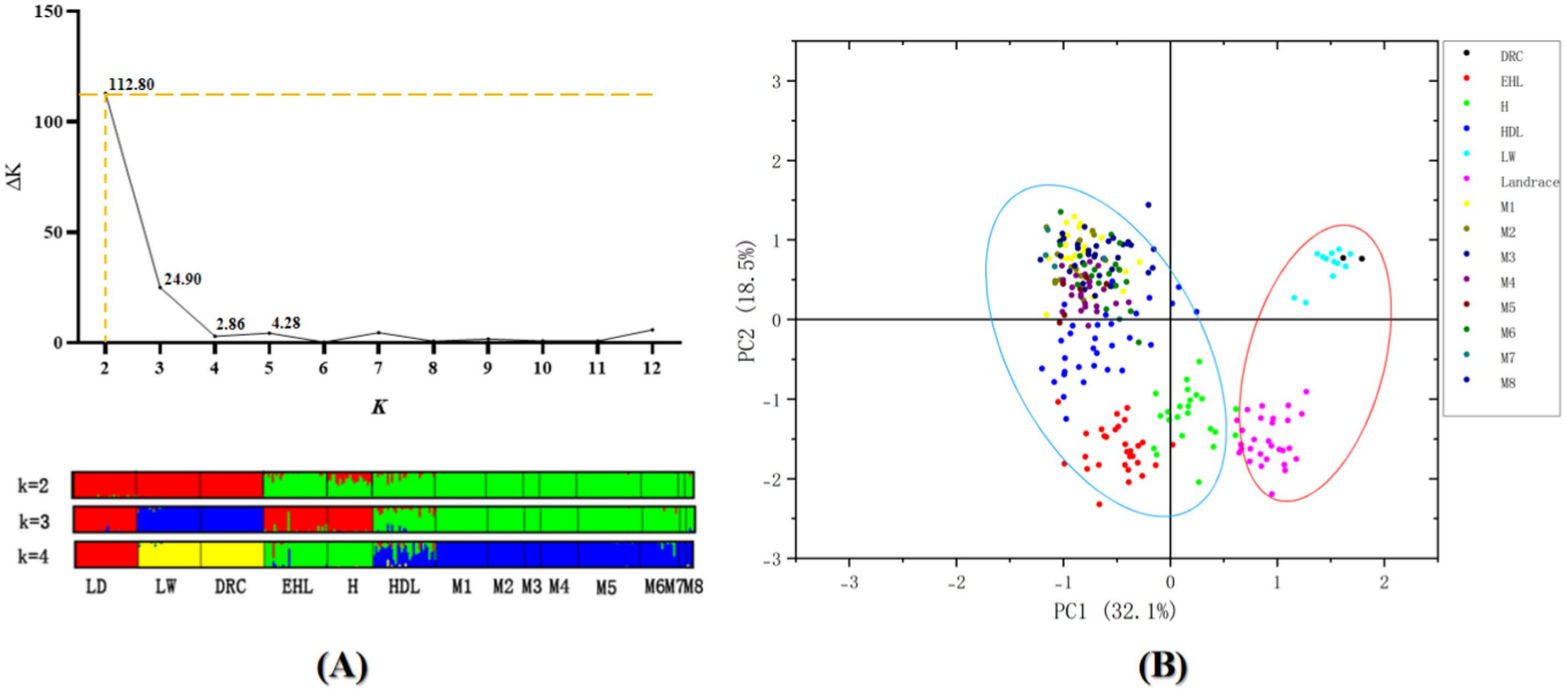
**(A)** Calculation of the true K of the YHSBLP following procedure outlined and graphical representation with K2-4 by STRUCTURE software. **(B)** PCA plot of Mi pig pedigree vs. other six breeds of pigs. LD, Landrace; LW, Large White; DRC, Duroc; EHL, Erhualian pig; HDL, Hongdenglong pig; M, Mi pig; H, Huai pig.

STRUCTURE analysis with the ΔK method suggested the population structure of four native breeds and three commercial breeds was optimum at K = 2 (Figure 3). Four breeds from the Jiangsu native population, including Erhualian, Huai, Hongdenglong, and Mi families, indicated that they shared one common ancestor, while Landrace, Large White, and Duroc pigs shared another. With the K value increased, Erhualian and Huai pigs showed a separation of Mi families with Hongdenglong pigs. PCA conducted through OriginPro 2024 comparing Mi populations with the other six breeds (Figure 5) revealed that five breeds including Landrace, Huai, Erhualian, Hongdenglong and Mi pigs clearly differentiated from each other, while there was no significant separation among Mi populations. In contrast, there was no significant separation between Duroc and Large White pigs. Both the Mi populations and the Hongdenglong pigs were relatively close to each other. These results indicated that the population distribution was generally consistent with the UPGMA tree.

## 4 Discussion

RIPs, originally proposed by Flavell (36), have been recognized as an effective molecular marker system for assessing phylogenetic relationships among primates and genetic relationships within human populations (37). Additionally, certain subfamilies of SINE are associated with specific lineages of bats (38). Clusters are effective in detecting the presence of different subpopulations within a breed (39). In our previous study, Mi pigs were divided into 8 populations, with each population generally corresponding to one family in the pedigree, as determined using SINE-RIPs (31). To accurately define the pedigree and enhance the conservation and breeding of Mi pigs, population structures were analyzed, and eight families were separated based on the UPGMA tree.

All eight Mi families exhibited genetic distances ranging from 0.3712 to 0.7609 with commercial breeds, and showed relatively closer distances ranging from 0.0943 to 0.6122 with native Jiangsu breeds. Within the Mi population itself, genetic distances varied from 0.0168 to 0.1340. The UPGMA tree reflected the same trends in genetic distances, illustrating strong genetic differentiation between the commercial breeds, Chinese native pigs, and Mi populations. Therefore, these eight families could be classified as eight lineages for Mi pigs, and this study carries significant implications for further breeding and conservation efforts concerning Mi pigs.

FIS is regarded as a measure of conservation priority because its values can indicate the level of endangerment and inbreeding within populations (40). Despite high genetic similarity among Mi populations, the FIS values for the eight Mi families were all below zero, suggesting that these families are outbreeding. However, the sizes of these families were uneven, with only 6, 5, and 7 individuals in families M5, M7, and M8 respectively, which poses a risk for ongoing conservation and increases susceptibility to inbreeding depression. At present, it is essential and urgent to expand population sizes and prevent inbreeding depression, by employing methods like those suggested by Zhao et al. (41).

Using SINE-RIP to assess genetic diversity, we observed that the PIC values among the seven breeds were low. According to the criteria suggested by Bostein et al. (42), all populations exhibited low polymorphism, with the exception of M8 and Erhualian, which were moderately polymorphic. Previous studies have similarly reported low PIC values in the populations examined (29, 30). In contrast, other molecular markers such as microsatellites typically showed relatively high PIC values, most of which exceeded 0.5 (43–45). Microsatellites were the most (48%) frequently used markers for genotyping local farm animal breeds (46) and are multi-allelic, whereas SINE-RIPs are bi-allelic. Therefore, the standards for evaluating low, moderate, or high polymorphic populations set by Botstein et al. may not be applicable to SINE-RIPs.

Most values of Ne and PIC for Mi populations and Jiangsu local breeds were higher than those for commercial pigs, suggesting that these Chinese native breeds possess greater genetic diversity compared to commercial pigs from breeding companies, which displayed lower levels of genetic diversity. The comparatively lower Ne and PIC values in Mi pigs relative to other native breeds imply that selective breeding during conservation efforts might have contributed to these reduced values.

The *F*-values (Fst) provide insights into the genetic diversity within breeds (39, 47). A Fst value less than 0.05 typically indicates minimal genetic differentiation among populations. This study revealed that the Fst values among the seven breeds were all above 0.05, with the greatest genetic distance observed between Duroc pigs and the other five breeds, highlighting significant genetic differentiation and rich genetic diversity among them. Between Mi families, the Fst values of M7 were relatively higher with other families from 0.1100 to 0.2265, probably because only five pigs are in this family, two of five be boars and there are no kinship with other families especially with M2.

Within the Jiangsu native breeds, Huai pigs are found in the northern region, whereas other native populations are located in the southern region; historical introgressions occurred among them hundreds of years ago (5). The UPGMA tree, STRUCTURE and PCA diagram showed that Huai, all Mi populations, with Hongdenglong, and Erhualian pigs, are markedly distinct from commercial pigs, and each native population exhibits its own degree of differentiation. While Erhualian and Hongdenglong pigs were originated from Mi pigs, Hongdenglong pigs were shown closer kinship than Erhualian pigs with Mi pigs. It is probably because the number of Erhualian pigs are much larger than those of Hongdenglong pigs and Mi pigs, while all Hongdenglong pigs and Mi pigs are almost kept in one conservation farm separately. Thus, expanding this study will enable the development of better conservation programs for the sustainable management of Mi pigs.

## 5 Conclusion

Based on SINE RIPs, Mi pigs were categorized into eight clusters, each containing one to three boars. All eight Mi families displayed significant genetic distance from commercial breeds while maintaining relatively close distances with Jiangsu native breeds, a trend confirmed by the UPGMA tree, STRUCTURE and PCA. The Mi populations were outbreeding and exhibited a degree of diversity as indicated by their FIS and Fst values, which contribute to the conservation efforts for Mi pigs. These findings confirm the utility of SINE RIPs in pig population structure and lay a theoretical foundation for the ongoing conservation and breeding efforts for Mi pigs.

## Data Availability

The datasets presented in this study can be found in online repositories. The names of the repository/repositories and accession number(s) can be found in the article/Supplementary material.

## References

[1] Zhang X. Comparison on partial traits and investigation on breed Resourses of Huai Bei Pig in Gan Yu region [Master].Nanjing: Nanjing Agricultural University. (2004).

[2] Cao HFHH Chen JF Jin WM Zheng QW. Mi Pig. Animal Husbandry Vet Med. (1988) 2:63–5.

[3] Jiajin Chen GZ Li Q. Research on the history and characteristics of germplasm resource of red lantern Pig. Ancient Modern Agricul. (2016) 4:68–79.

[4] Chang Q Zhou KY Wang YQ Zhang ZK Cao X. Rapd analysis of genetic diversity and phylogenetic relationship of the Taihu pigs. Yi Chuan Xue Bao. (1999) 26:480–8. 10665224

[5] Fan B Wang ZG Li YJ Zhao XL Liu B Zhao SH. Genetic variation analysis within and among Chinese indigenous swine populations using microsatellite markers. Anim Genet. (2002) 33:422–7. doi: 10.1046/j.1365-2052.2002.00898.x 12464016

[6] FAO. The state of the World’s biodiversity for food and agriculture, vol. 2019. Rome, Italy: FAO (2019).

[7] Groenen MAM Archibald AL Uenishi H Tuggle CK Takeuchi Y Rothschild MF. Analyses of Pig genomes provide insight into porcine demography and evolution. Nature. (2012) 491:393–8. doi: 10.1038/nature11622, PMID: 23151582 PMC3566564

[8] Chen C Wang W Wang X Shen D Wang S Wang Y. Retrotransposons evolution and impact on Lncrna and protein coding genes in pigs. Mob DNA. (2019) 10:19. doi: 10.1186/s13100-019-0161-8, PMID: 31080521 PMC6501411

[9] Zhao PJ Gu LH Gao YH Pan ZY Liu L Li XZ. Young Sines in Pig genomes impact gene regulation, genetic diversity, and complex traits. Commun Biol. (2023) 6:894. doi: 10.1038/s42003-023-05234-x, PMID: 37652983 PMC10471783

[10] Platt RN Vandewege MW Ray DA. Mammalian transposable elements and their impacts on genome evolution. Chromosom Res. (2018) 26:25–43. doi: 10.1007/s10577-017-9570-z, PMID: 29392473 PMC5857283

[11] Kalendar R Flavell AJ Ellis THN Sjakste T Moisy C Schulman AH. Analysis of plant diversity with retrotransposon-based molecular markers. Heredity. (2011) 106:520–30. doi: 10.1038/hdy.2010.93, PMID: 20683483 PMC3183911

[12] Göke J Ng HH. Ctrl plus insert: retrotransposons and their contribution to regulation and innovation of the transcriptome. EMBO Rep. (2016) 17:1131–44. doi: 10.15252/embr.201642743, PMID: 27402545 PMC4967949

[13] Wang XY Chi CL He J Du ZY Zheng Y D'Alessandro E. Sine insertion may act as a repressor to affect the expression of Pig and growth traits. Genes-Basel. (2022) 13:1422. doi: 10.3390/genes13081422, PMID: 36011333 PMC9407865

[14] Chi CL He J Du ZY Zheng Y D'Alessandro E Chen C. Two retrotransposon elements in intron of porcine is associated with phenotypic variation. Life-Basel. (2022) 12:1650. doi: 10.3390/life12101650, PMID: 36295085 PMC9604734

[15] Chen C Zheng Y Wang ML Murani E D'Alessandro E Moawad AS. Sine insertion in the intron of Pig Ghr may decrease its expression by acting as a repressor. Animals-Basel. (2021) 11:1871. doi: 10.3390/ani11071871, PMID: 34201672 PMC8300111

[16] Zheng Y Chen C Wang ML Moawad AS Wang XY Song CY. Sine insertion in the Pig carbonic anhydrase 5b gene is associated with changes in gene expression and phenotypic variation. Animals-Basel. (2023) 13:1942. doi: 10.3390/ani13121942, PMID: 37370452 PMC10295633

[17] Shedlock AM Takahashi K Okada N. Sines of speciation: tracking lineages with Retroposons. Trends Ecol Evol. (2004) 19:545–53. doi: 10.1016/j.tree.2004.08.002, PMID: 16701320

[18] Seibt KM Wenke T Wollrab C Junghans H Muders K Dehmer KJ. Development and application of Sine-based markers for genotyping of potato varieties. Theor Appl Genet. (2012) 125:185–96. doi: 10.1007/s00122-012-1825-7, PMID: 22371142

[19] Wenke T Seibt KM Dobel T Muders K Schmidt T. Inter-Sine amplified polymorphism (Isap) for rapid and robust plant genotyping. Methods Molecular Biol (Clifton, N J). (2015) 1245:183–92. doi: 10.1007/978-1-4939-1966-6_14, PMID: 25373758

[20] Hasan N Choudhary S Naaz N Sharma N Laskar RA. Recent advancements in molecular marker-assisted selection and applications in plant breeding Programmes. J Genet Eng Biotechn. (2021) 19:128. doi: 10.1186/s43141-021-00231-1, PMID: 34448979 PMC8397809

[21] Baloch FS Altaf MT Liaqat W Bedir M Nadeem MA Cömertpay G. Recent advancements in the breeding of Sorghum crop: current status and future strategies for marker-assisted breeding. Front Genet. (2023) 14:14. doi: 10.3389/fgene.2023.1150616, PMID: 37252661 PMC10213934

[22] Batzer MA Stoneking M Alegria-Hartman M Bazan H Kass DH Shaikh TH. African origin of human-specific polymorphic Alu insertions. Proc Natl Acad Sci USA. (1994) 91:12288–92. doi: 10.1073/pnas.91.25.12288, PMID: 7991620 PMC45422

[23] Di Santo Meztler GP Del Palacio S Esteban ME Armoa I Argüelles CF Catanesi CI. Genetic differentiation of north-East Argentina populations based on 30 binary X chromosome markers. Front Genet. (2018) 9:208. doi: 10.3389/fgene.2018.00208, PMID: 29951085 PMC6008373

[24] Shimamura M Yasue H Ohshima K Abe H Kato H Kishiro T. Molecular evidence from Retroposons that whales form a clade within even-toed ungulates. Nature. (1997) 388:666–70. doi: 10.1038/41759, PMID: 9262399

[25] Liu D Li Y Tang W Yang J Guo H Zhu G. Population structure of *Coilia Nasus* in the Yangtze River revealed by insertion of short interspersed elements. Biochem Syst Ecol. (2014) 54:103–12. doi: 10.1016/j.bse.2013.12.022

[26] Chessa B Pereira F Arnaud F Amorim A Goyache F Mainland I. Revealing the history of sheep domestication using retrovirus integrations. Science. (2009) 324:532–6. doi: 10.1126/science.1170587, PMID: 19390051 PMC3145132

[27] Lee J Mun S Kim DH Cho CS Oh DY Han K. Chicken (Gallus Gallus) endogenous retrovirus generates genomic variations in the chicken genome. Mob DNA. (2017) 8:2. doi: 10.1186/s13100-016-0085-5, PMID: 28138342 PMC5260121

[28] Chen C D'Alessandro E Murani E Zheng Y Giosa D Yang NS. Sine jumping contributes to large-scale polymorphisms in the Pig genomes. Mobile DNA-Uk. (2021) 12:17. doi: 10.1186/s13100-021-00246-y PMC8240389 34183049

[29] Chen C Wang XY Zong WC D'Alessandro E Giosa D Guo YF. Genetic diversity and population structures in Chinese miniature pigs revealed by Sine retrotransposon insertion polymorphisms, a new type of genetic markers. Animals-Basel. (2021) 11:1136. doi: 10.3390/ani11041136, PMID: 33921134 PMC8071531

[30] Wang XY D'Alessandro E Chi CL Moawad AS Zong WC Chen C. Genetic evaluation and population structure of Jiangsu native pigs in China revealed by Sine insertion polymorphisms. Animals. (2022) 12:1345. doi: 10.3390/ani12111345, PMID: 35681812 PMC9179424

[31] Chenlin Chi LN Tao Y Chen C Zhou C Song C Wang X. Population structure and genetic diversity analysis of Sujiang pigs based on Sine-Rips markers. Chinese J Anim Sci. (2023) 52:147–54. doi: 10.19556/j.0258-7033.20211213-09

[32] Yeh F Yang RC Boyle T. Popgene version 1.32 Microsoft windows-based freeware for populations genetic analysis. Edmonton: University of Alberta (1999).

[33] Serrote CML Reiniger LRS Silva KB Rabaiolli S Stefanel CM. Determining the polymorphism information content of a molecular marker. Gene. (2020) 726:144175. doi: 10.1016/j.gene.2019.144175, PMID: 31726084

[34] Kumar S Stecher G Tamura K. Mega7: molecular evolutionary genetics analysis version 7.0 for bigger datasets. Mol Biol Evol. (2016) 33:1870–4. doi: 10.1093/molbev/msw054, PMID: 27004904 PMC8210823

[35] Seifert E. Originpro 9.1: scientific data analysis and graphing software-software review. J Chem Inf Model. (2014) 54:1552. doi: 10.1021/ci500161d, PMID: 24702057

[36] Flavell AJ Knox MR Pearce SR Ellis TH. Retrotransposon-based insertion polymorphisms (Rbip) for high throughput marker analysis. Plant J. (1998) 16:643–50. doi: 10.1046/j.1365-313x.1998.00334.x, PMID: 10036780

[37] Uchida M Li XW Mertens P Alpar HO. Transfection by particle bombardment: delivery of plasmid DNA into mammalian cells using gene gun. Biochim Biophys Acta. (2009) 1790:754–64. doi: 10.1016/j.bbagen.2009.05.013, PMID: 19477233

[38] Ray DA Pagan HJT Platt RN Kroll AR Schaack S Stevens RD. Differential Sine evolution in vesper and non-vesper bats. Mobile DNA-Uk. (2015) 6:10. doi: 10.1186/s13100-015-0038-4, PMID: 25991928 PMC4436864

[39] Toro MA Fernández J Caballero A. Molecular characterization of breeds and its use in conservation. Livest Sci. (2009) 120:174–95. doi: 10.1016/j.livsci.2008.07.003

[40] Liu G Zhao QJ Lu J Sun FZ Han X Zhao JJ. Insights into the genetic diversity of indigenous goats and their conservation priorities. Asian Austral J Anim. (2019) 32:1501–10. doi: 10.5713/ajas.18.0737, PMID: 30744325 PMC6718908

[41] Zhao X Zhu M Pan Y Hou Q Ni L. Protection, development and utilization of local Pig genetic resources in Jiangsu Province (in Chinese). Jiangsu Agricul Sci. (2018) 46:179–81. doi: 10.15889/j.issn.1002-1302.2018.19.048

[42] Botstein D White RL Skolnick M Davis RW. Construction of a genetic linkage map in man using restriction fragment length polymorphisms. Am J Hum Genet. (1980) 32:314–31. PMID: 6247908 PMC1686077

[43] Djimènou D Adoukonou-Sagbadja H Dayo GK Chrysostome CAAM Koudande DO. Genetic diversity and phylogenetic relationships within local pigs in southern Benin. Trop Anim Health Prod. (2021) 53:434. doi: 10.1007/s11250-021-02857-2, PMID: 34387779

[44] Fabuel E Barragán C Silió L Rodríguez MC Toro MA. Analysis of genetic diversity and conservation priorities in Iberian pigs based on microsatellite markers. Heredity. (2004) 93:104–13. doi: 10.1038/sj.hdy.6800488, PMID: 15150539

[45] Sahoo NR Nesa N Naskar S Banik S Pankaj PK Sahoo M. Microsatellite and mitochondrial diversity analysis of native pigs of indo-Burma biodiversity hotspot. Anim Biotechnol. (2016) 27:52–9. doi: 10.1080/10495398.2015.1083030, PMID: 26695527

[46] Olschewsky A Hinrichs D. An overview of the use of genotyping techniques for assessing genetic diversity in local farm animal breeds. Animals-Basel. (2021) 11:2016. doi: 10.3390/ani11072016, PMID: 34359144 PMC8300386

[47] Caballero A García-Dorado A. Allelic diversity and its implications for the rate of adaptation. Genetics. (2013) 195:1373–84. doi: 10.1534/genetics.113.158410, PMID: 24121776 PMC3832279

[48] Committee TPB. Chinese Taihu Pig: Chinese Taihu Pig Committee TPB. Shanghai Science and Technology Press. (1991).

