# Supplementary material for: Population structure and genetic diversity of Mi pigs based on SINE-RIPs
Source: Front Vet Sci. 2025 Apr 24;12:1500115. doi: 10.3389/fvets.2025.1500115 (PMC12058712; doi:10.3389/fvets.2025.1500115)
Supplement: Supplementary file 1 [file Table_1.docx]

**Table：**

**Table 1 : Analysis of SINE-RIPs Polymorphism in Mi Pig.**

| RIP | Insertion Frequency | | | | | | | | | | | | | | | Number of Populations without Polymorphism | Number of Populations with Polymorphism / Number of Populations with Hardy-Weinberg Imbalance Violation |
| --- | --- | --- | --- | --- | --- | --- | --- | --- | --- | --- | --- | --- | --- | --- | --- | --- | --- |
|  | LD | LW | DRC | EHL | H | HDL | M（average） | M1 | M2 | M3 | M4 | M5 | M6 | M7 | M8 |  |  |
| ESA1-98 | 1.00 | - | - | 1.00 | - | 0.20 | 0.36 | 0.10 | 0.75 | 0.26 | - | 0.58 | 0.24 | - | 0.21 | 7 | 8/0 |
| REF-12270 | 0.44 | 0.45 | - | 0.48 | - | 0.77 | 0.68 | 0.96 | 0.64 | 0.43 | 0.71 | 1.00 | 0.89 | 0.70 | 0.14 | 3 | 12/4 |
| REF-13182 | - | 0.08 | 0.19 | 1.00 | 1.00 | 0.83 | 0.99 | 1.00 | 1.00 | 1.00 | 0.98 | 1.00 | 1.00 | 1.00 | 0.93 | 9 | 6/0 |
| REF-14427 | - | - | - | 0.58 | 0.96 | 0.52 | 0.26 | 0.10 | - | 0.37 | 0.31 | 0.42 | 0.11 | - | - | 6 | 9/2 |
| REF-16131 | - | - | - | 0.39 | 0.22 | - | 0.63 | 0.21 | 0.72 | 0.63 | 0.71 | 0.83 | 0.71 | 0.75 | 0.50 | 4 | 11/3 |
| REF-16266 | - | - | - | 0.08 | - | 0.81 | 0.62 | 0.70 | 0.69 | 0.69 | 0.23 | 0.50 | 0.61 | 0.70 | 0.86 | 4 | 11/2 |
| REF-17668 | - | - | - | 0.39 | - | - | 0.10 | 0.08 | 0.03 | 0.07 | - | 0.33 | 0.03 | - | 0.07 | 7 | 8/1 |
| ESA2-58 | 0.67 | 0.03 | - | 0.92 | 0.43 | 0.61 | 0.40 | 0.19 | 0.19 | 0.13 | 0.56 | 0.33 | - | 1.00 | - | 3 | 12/2 |
| REF-21609 | 0.13 | - | - | 0.05 | 0.13 | 0.55 | 0.54 | 0.62 | 0.53 | 0.67 | 0.24 | 0.33 | 0.29 | 0.90 | 0.71 | 2 | 13/3 |
| REF-2929 | 0.44 | 0.03 | - | 0.84 | 0.67 | 0.59 | 0.53 | 0.65 | 0.67 | 0.56 | 0.54 | 0.67 | 0.39 | 0.30 | 0.43 | 1 | 14/6 |
| ESA1-16 | 1.00 | 0.16 | - | 0.98 | 0.37 | 0.81 | 0.82 | 1.00 | 1.00 | 0.94 | 0.94 | 0.58 | 0.82 | 0.80 | 0.50 | 4 | 11/0 |
| REF-3992 | 0.63 | - | - | 0.59 | 1.00 | 0.58 | 0.11 | 0.02 | - | 0.06 | - | - | 0.21 | - | 0.14 | 6 | 9/1 |
| REF-5597 | - | 0.03 | - | 0.52 | - | 0.91 | 0.88 | 0.88 | 1.00 | 0.96 | 1.00 | 0.83 | 0.64 | 1.00 | 0.71 | 6 | 9/1 |
| ESA2-18 | 0.64 | - | - | 0.66 | 0.43 | 0.64 | 0.15 | 0.12 | 0.03 | 0.06 | 0.38 | 0.17 | 0.21 | 0.10 | 0.14 | 2 | 13/1 |
| ESA1-33 | 1.00 | - | - | 0.23 | 0.39 | 0.09 | 0.18 | 0.04 | - | 0.07 | - | - | 0.24 | - | 0.36 | 7 | 8/1 |
| REF-9435 | - | - | - | 0.84 | 0.61 | 0.81 | 0.99 | 1.00 | 1.00 | 0.98 | 1.00 | 1.00 | 1.00 | 1.00 | 0.93 | 9 | 6/0 |
| REF-10096 | 0.08 | 0.08 | - | 0.53 | 0.89 | 0.53 | 0.38 | 0.31 | 0.03 | 0.41 | 0.52 | 0.50 | 0.66 | 0.50 | 0.14 | 1 | 14/6 |
| REF-11062 | 0.40 | - | - | 0.45 | 0.63 | 0.80 | 0.91 | 0.96 | 1.00 | 0.89 | 0.98 | 0.92 | 0.89 | 0.80 | 0.86 | 3 | 12/1 |
| Number of Non-Polymorphic Sites in a Population | 7 | 11 | 17 | 0 | 5 | 2 | - | - | 8 | 1 | 6 | 5 | 3 | 9 | 2 | - | - |

**Table 2 : Genetic Parameters of Mi populations and Six Breeds by 18 SINE-RIPs.**

| Breeds or population | number | Ho | He | Ne | Fis | PIC |
| --- | --- | --- | --- | --- | --- | --- |
| LD | 32 | 0.7760±0.3120 | 0.8191±0.2262 | 1.3249±0.4257 | -0.2192±0.3907 | 0.1509±0.1691 |
| LW | 32 | 0.9323±0.1190 | 0.9306±0.1312 | 1.1039±0.2387 | -0.0387±0.1027 | 0.0745±0.1191 |
| DRC | 32 | 0.9792±0.0884 | 0.9828±0.0730 | 1.0243±0.1033 | -0.2308±0.0000 | 0.0331±0.0982 |
| EHL | 32 | 0.6892±0.2347 | 0.6805±0.1999 | 1.5687±0.4102 | 0.0070±0.2802 | 0.2532±0.1393 |
| H | 23 | 0.7536±0.2400 | 0.7643±0.2243 | 1.4123±0.4214 | -0.0708±0.1428 | 0.1912±0.1940 |
| HDL | 32 | 0.6337±0.1993 | 0.6645±0.1643 | 1.5699±0.3446 | -0.1056±0.2261 | 0.2690±0.1149 |
| M1 | 26 | 0.7617±0.2191 | 0.7975±0.1690 | 1.3068±0.3063 | -0.1428±0.1570 | 0.1767±0.1293 |
| M2 | 19 | 0.7716±0.2889 | 0.8240±0.2117 | 1.2936±0.3742 | -0.2421±0.2240 | 0.1488±0.1602 |
| M3 | 27 | 0.6847±0.2789 | 0.7514±0.1861 | 1.4073±0.3738 | -0.1890±0.2301 | 0.2075±0.1323 |
| M4 | 26 | 0.6674±0.3397 | 0.7666±0.2232 | 1.4069±0.4144 | -0.3504±0.2472 | 0.1901±0.1643 |
| M5 | 6 | 0.5556±0.3524 | 0.6835±0.2274 | 1.5207±0.4088 | -0.4744±0.2549 | 0.2351±0.1520 |
| M6 | 19 | 0.6363±0.2604 | 0.7150±0.1773 | 1.4562±0.3250 | -0.2617±0.2324 | 0.2326±0.1297 |
| M7 | 5 | 0.7778±0.3059 | 0.8058±0.2169 | 1.2863±0.3428 | -0.2231±0.5003 | 0.1532±01545 |
| M8 | 7 | 0.6905±0.2558 | 0.7027±0.1729 | 1.4500±0.3349 | -0.0847±0.4641 | 0.2501±0.1064 |
| Average | - | 0.7364±0.1095 | 0.7777±0.0895 | 1.3666±0.1534 | -0.1876±0.1237 | 0.1833±0.0655 |

**Table 3 : Nei's Genetic Identity and Genetic Distances between Mi populations and Six Breeds.**

Note: “-” indicated diagonal. Nei's genetic identity is presented above the diagonal, and genetic distance is presented below the diagonal. LD: Landrace; LW: Large White; DRC: Duroc; EHL: Erhualian; HDL: Hongdenglong; M1-8: Mi population; H: Huai pig.

| ID | LD | LW | DRC | EHL | H | HDL | M1 | M2 | M3 | M4 | M5 | M6 | M7 | M8 |
| --- | --- | --- | --- | --- | --- | --- | --- | --- | --- | --- | --- | --- | --- | --- |
| LD | - | 0.7306 | 0.7039 | 0.7336 | 0.6303 | 0.6116 | 0.5299 | 0.5509 | 0.5232 | 0.5320 | 0.5043 | 0.5614 | 0.4673 | 0.5718 |
| LW | 0.3139 | - | 0.9858 | 0.5368 | 0.6484 | 0.5898 | 0.6240 | 0.5709 | 0.6163 | 0.6123 | 0.5860 | 0.6501 | 0.5607 | 0.6899 |
| DRC | 0.3511 | 0.0143 | - | 0.5171 | 0.6674 | 0.5530 | 0.5794 | 0.5483 | 0.6028 | 8.5792 | 0.5445 | 0.6104 | 0.5326 | 0.6842 |
| EHL | 0.3098 | 0.6221 | 0.6596 | - | 0.7769 | 0.8095 | 0.7170 | 0.7504 | 0.7261 | 0.7964 | 0.8049 | 0.7460 | 0.6935 | 0.7195 |
| H | 0.4616 | 0.4332 | 0.4043 | 0.2525 | - | 0.7474 | 0.6156 | 0.5422 | 0.6386 | 0.6996 | 0.6417 | 0.6975 | 0.5814 | 0.6981 |
| HDL | 0.4917 | 0.5280 | 0.5925 | 0.2113 | 0.2911 | - | 0.9100 | 0.8255 | 0.8670 | 0.8821 | 0.8437 | 0.8638 | 0.8591 | 0.8735 |
| M1 | 0.6351 | 0.4716 | 0.5458 | 0.3326 | 0.4851 | 0.0943 | - | 0.9378 | 0.9619 | 0.9283 | 0.9169 | 0.9428 | 0.9073 | 0.9552 |
| M2 | 0.5961 | 0.5605 | 0.6008 | 0.2872 | 0.6122 | 0.1918 | 0.0642 | - | 0.9656 | 0.8976 | 0.9318 | 0.9146 | 0.8793 | 0.9437 |
| M3 | 0.6478 | 0.4840 | 0.5062 | 0.3201 | 0.4484 | 0.1427 | 0.0388 | 0.0350 | - | 0.9312 | 0.9199 | 0.9489 | 0.9243 | 0.9833 |
| M4 | 0.6311 | 0.4905 | 0.5460 | 0.2276 | 0.3573 | 0.1255 | 0.0744 | 0.1080 | 0.0713 | - | 0.9320 | 0.9329 | 0.9213 | 0.9216 |
| M5 | 0.6846 | 0.5345 | 0.6079 | 0.2170 | 0.4436 | 0.1700 | 0.0868 | 0.0706 | 0.0835 | 0.0704 | - | 0.9415 | 0.8755 | 0.9101 |
| M6 | 0.5773 | 0.4306 | 0.4937 | 0.2931 | 0.3602 | 0.1464 | 0.0589 | 0.0893 | 0.0524 | 0.0695 | 0.0603 | - | 0.8746 | 0.9503 |
| M7 | 0.7609 | 0.5786 | 0.6300 | 0.3660 | 0.5424 | 0.1519 | 0.0973 | 0.1287 | 0.0787 | 0.0819 | 0.1329 | 0.1340 | - | 0.9030 |
| M8 | 0.5589 | 0.3712 | 0.3795 | 0.3292 | 0.3593 | 0.1353 | 0.0458 | 0.0579 | 0.0168 | 0.0817 | 0.0942 | 0.0510 | 0.1021 | - |

**Table S1:** **Primer for 18 SINE-RIPs.**

|  | Loci | Primer Sequence | Location | Annealing temperature（℃） | PCR Product (bp) |
| --- | --- | --- | --- | --- | --- |
| 1 | ESA1-98 | F-GAGCATTGCACCTGCACTTA  R-CACTTCTTGCAAAACACTGTAGG | chr1:119154448-119155108 | 58 | 404/661 |
| 2 | REF-12270 | F-CTTTTGTCCTTCACTGTTCATCA  R-TCTGCGTTGTGTCCACTCTA | chr2:132104503-132104952 | 58 | 450/713 |
| 3 | REF-13182 | F-GGAGGCAAAGGGAAAGAGTC  R-GCTCCCATTCCTGTTGTTGT | chr3:74208760-74209181 | 58 | 432/727 |
| 4 | REF-14427 | F-ACAACACAAGCCCCAAATGA  R-TGACTTTTCTGTGTTGGTCTTGT | chr4:79318193-79318592 | 58 | 400/650 |
| 5 | REF-16131 | F-TGCTTTTCTTCTGGGAGGTG  R-CAACGCTTGCCAGATTTTCT | chr5:98261686-98262086 | 58 | 432/732 |
| 6 | REF-16266 | F-TCCGCCCATCTGAATAATAA  R-GCATTACCTTTGGTGAGAGG | chr6:9549227-9549524 | 58 | 298/555 |
| 7 | REF-17668 | F-CCTTTGCCATTTCTCCAGTA  R-AGTTACAGACCCGGCTTACC | chr7:1569064-1569342 | 58 | 279/561 |
| 8 | ESA2-58 | F-GGAGCCTTTCTGTGTTTCCA  R-GAGCAAGAAGAGGGGAGGTAA | chr8：28073574-28074197 | 58 | 361/624 |
| 9 | REF-21609 | F-GAATGCCACTTTCCCCACAA  R-TCAGTGAGTAGGTGGCAGAG | chr9：122993311-122993721 | 58 | 411/693 |
| 10 | REF-2929 | F-CCAACTGCATGCTCTTCCAG  R-TCCTGATTATCTTGGAAATGGCT | chr10:59802500-59802944 | 58 | 445/739 |
| 11 | ESA1-16 | F-GTCCCCAAAACAGTGTGGAG  R-ACTCGGGGAACTTCCTTCAT | chr11:68768136-68768788 | 58 | 395/653 |
| 12 | REF-3992 | F-ATGTACCCTCCCCTCAAATC  R-TACCCCAAACACCAAAACAT | chr12:3226907-3227211 | 58 | 305/603 |
| 13 | REF-5597 | F-AACCAAAGCAGTGTTCAGGG  R-AGGGTTTGGGATGATGATGGT | chr13:106226070-106226486 | 58 | 417/710 |
| 14 | ESA2-18 | F-AGTCCTGTGCTGGGAAGTTG  R-TTCCACTTTGCAGCATTTTG | chr14:107388631-107389282 | 58 | 385/652 |
| 15 | ESA1-33 | F-TCTGATAGGCGCTGGATCTT  R-GGCCCTGGAGCTTCTACAT | chr15:104894531-104895172 | 58 | 385/642 |
| 16 | REF-9432 | F-TGGCTGTAGGATTGTGACTG  R-TTGGTGAAAATCCAAAACCT | chr16:55338119-55338391 | 58 | 273/495 |
| 17 | REF-10096 | F-CTCGCCCCTTACTTCAGACA  R-AACCATCACCACTGAACCCC | chr17:30484896-30485305 | 58 | 410/672 |
| 18 | REF-11062 | F-AGTCTCCCACTCACATTGCC  R-CCTCTGAGCTGCTCTTCCTT | chr18:50578007-50578406 | 58 | 400/690 |
